# Sub-100-nm ordered silicon hole arrays by metal-assisted chemical etching

**DOI:** 10.1186/1556-276X-8-410

**Published:** 2013-10-04

**Authors:** Hidetaka Asoh, Kousuke Fujihara, Sachiko Ono

**Affiliations:** 1 Department of Applied Chemistry, Faculty of Engineering, Kogakuin University, 2665-1 Nakano, Hachioji, Tokyo 192-0015, Japan

**Keywords:** Silicon nanohole, Electroless deposition, Noble metal dot arrays, Metal-assisted chemical etching, High aspect ratio, Anodic porous alumina

## Abstract

Sub-100-nm silicon nanohole arrays were fabricated by a combination of the site-selective electroless deposition of noble metals through anodic porous alumina and the subsequent metal-assisted chemical etching. Under optimum conditions, the formation of deep straight holes with an ordered periodicity (e.g., 100 nm interval, 40 nm diameter, and high aspect ratio of 50) was successfully achieved. By using the present method, the fabrication of silicon nanohole arrays with 60-nm periodicity was also achieved.

## Background

Silicon has attracted attention as the most important material for the semiconductor industry. Various techniques such as reactive ion etching, electrochemical etching, and anisotropic chemical etching are used in fabricating silicon-based functional devices [1]. Among them, metal-assisted chemical etching, which was proposed by Li and Bohn in 2000 [2], has also attracted attention as a key nanofabrication method owing to its relative simplicity and low cost.

In general, metal-assisted chemical etching proceeds by immersing a silicon substrate decorated with a noble metal in an etchant composed of HF and an oxidative agent such as H_2_O_2_. To form metal catalytic layers on a silicon substrate with or without pattern regularity, physical deposition techniques in vacuum such as focused ion beam deposition [3], sputtering [2,4,5], conventional vacuum vapor deposition [6], and electron beam evaporation [7] are generally used. Because the morphology of the resultant silicon structures depends on the initial geometric pattern and dimensions of the noble metal catalyst, it is essential to use a patterned metal catalyst for the fabrication of ordered silicon nanostructures. For example, if a metal catalytic layer with an ordered pore arrangement is applied, the silicon substrate is etched into an array of silicon nanowires. In 2007, Huang et al. demonstrated that silicon nanowires with an aspect ratio larger than 30 could be obtained using nanosphere lithography-based metal-assisted chemical etching [8]. For an overview of the fabrication of silicon by metal-assisted chemical etching, see review papers [9,10].

Until now, we have focused on the direct patterning of metal catalysts using a mask without the use of conventional lithographic techniques and reported the fabrication of ordered silicon micro-hole arrays by metal-assisted chemical etching using noble metal thin film arrays formed by sputtering through a polymer mask with micrometer openings [11-14]. In these cases, however, the periodicity and diameter of the obtained silicon hole arrays were limited to the micrometer order because the preparation of the polymer mask was based on colloidal crystal templating using microspheres. Although the fabrication of silicon hole arrays with a 200-nm periodicity was achieved using polystyrene nanospheres as an indirect mask in our other approach [15], further miniaturization of hole periodicity remains one of the significant projects. Therefore, the fabrication of silicon nanohole arrays with a periodicity of less than 100 nm by a similar strategy using another structural control material, which is formed by self-organization, to replace a polymer mask or colloidal crystals is required.

With the above background, anodic porous alumina, which has a typical naturally occurring self-ordered porous structure on the nanometer scale, is a candidate mask material for the fabrication of ordered silicon nanostructures using metal-assisted chemical etching. Huang et al. previously reported the successful etching of a silicon substrate into nanowires with diameters less than 10 nm using an ultrathin anodic alumina mask to pattern a noble metal mesh [4]. However, their approach shows difficulty in handling an alumina mask with a thickness of less than 100 nm. It is thus important to develop a versatile method that requires no specialized skills for preparing alumina masks. Except for anodic alumina mask, we fabricated silicon nanohole arrays with single pore diameters in the 10-nm range using a self-aligned block copolymer Au nanoparticle template [16]. However, further study on the effect of etching conditions (e.g., etching time and noble metal catalyst species) on the morphology of etched silicon in the sub-100-nm size scale, especially hole depth and aspect ratio, was needed.

Regarding fabrication of silicon nanohole arrays using electrochemical process, we tried previously to fabricate ordered nanohole arrays with high aspect ratio structures onto a silicon substrate using a combined process of electrochemical formation of porous alumina mask on a silicon substrate and electrochemical etching of silicon through the pores of alumina mask [17]. Although selective chemical etching of exposed silicon could be achieved, the resulting hole arrays were extremely shallow holes. Zacharatos et al. demonstrated that the fabrication of ordered nanostructures on the silicon surface could be achieved by a similar process [18]. However, the obtained hole structures were also shallow hole arrays. According to their report, the depth and aspect ratio of the silicon holes using oxalic acid for alumina mask formation were approximately 300 nm and approximately 1.5, respectively. When sulfuric acid was applied for anodization, the depth and aspect ratio of the silicon holes were 30 to 100 nm and approximately 2.5, respectively [18]. In 2009, the same group reported that macroporous silicon with an aspect ratio of 5.5 could be obtained on p-type silicon substrate using similar nonlithographic approach [19]. The pore diameter and pore depth of porous silicon were 180 nm and approximately 1 μm, respectively. Eventually, it was difficult to fabricate the ordered silicon nanohole arrays with a depth of more than 1 μm using electrochemical etching through anodic alumina mask.

In this study, we prepared a porous alumina mask directly on a silicon substrate by anodizing an aluminum film sputtered on silicon. Using localized metal deposition and the subsequent metal-assisted chemical etching through the porous alumina mask, we demonstrated that ordered nanohole arrays on a silicon substrate with a periodicity of less than 100 nm could be fabricated without the use of conventional lithographic techniques. The main purpose of our present study is to propose a new fabrication method of silicon nanohole array with a high aspect ratio by metal-assisted chemical etching without applying an external bias. In addition, we investigated the effect of noble metal catalyst species on the morphology of etched silicon.

## Methods

**Figure 1:**
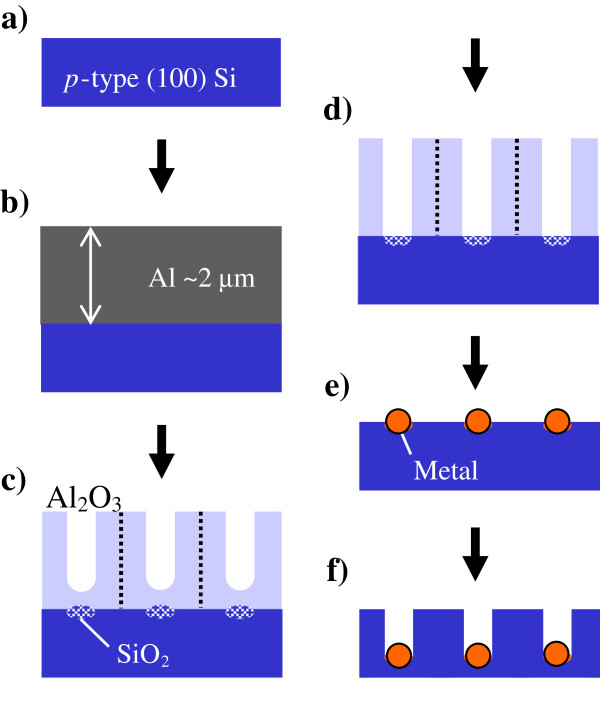
**Schematic model of fabrication of Si nanohole arrays. (a)** Si substrate, **(b)** aluminum film sputtered on Si substrate, **(c)** localized anodization of Si surface through barrier layer of upper porous alumina, **(d)** removal of barrier layer by chemical etching in phosphoric acid, **(e)** electroless plating, and **(f)** chemical etching of Si using Ag particles as catalyst.

The principle of the fabrication of silicon nanohole arrays by metal-assisted chemical etching is schematically shown in Figure 1. An approximately 2-μm-thick aluminum film was produced by DC sputtering (Shinko-Seiki SDM4314) on a p-type Si substrate (B-doped, 0.013 to 0.02 Ω cm, (100) crystal orientation) (Figure 1a,b). The pressure of the sputtering gas during deposition was 4.0 × 10^-1^ Pa. The sputtering power was 2 kW, and the deposition rate was approximately 4 nm s^-1^. After annealing at 300°C in air for 3 h to remove mechanical stress, the aluminum film sputtered on the silicon substrate was anodized at a constant voltage of 40 V in 0.3 mol dm^-3^ oxalic acid at 20°C (Figure 1c) [20,21]. These anodization conditions are well known as typical self-ordering conditions for forming highly ordered pore arrays in anodic alumina. The formation behavior of anodic porous alumina on the silicon substrate was examined by measuring current density transient at a constant voltage. After anodization, the anodized specimens were immersed in 5 wt.% phosphoric acid at 25°C for 10 min to remove the barrier layer of the anodic porous alumina (Figure 1d). The periodicity of the pores in the alumina mask used for the localized metal deposition described below was basically determined by the anodization voltage under appropriate anodization conditions. In this work, anodization at 25 V in 0.3 mol dm^-3^ sulfuric acid at 20°C was also conducted to prepare an ordered porous alumina mask with an approximately 60-nm periodicity [22].

The transfer of a nanoporous pattern of anodic porous alumina into a silicon substrate was attempted to etch the silicon substrate by metal-assisted chemical etching. First, electroless plating was used to form a metal catalyst pattern on silicon. In the case of the Ag catalyst, anodized silicon with a porous alumina mask was immersed in a solution of 2 × 10^-3^ mol dm^-3^ AgNO_3_ and 5 mol dm^-3^ HF for 15 s (Figure 1e). In the case of Au deposition, the specimens were immersed in a solution of 2 × 10^-3^ mol dm^-3^ Na[AuCl_4_] · 2H_2_O and 5 mol dm^-3^ HF for 15 s. After metal deposition, the silicon substrate was chemically etched in 5 mol dm^-3^ HF containing 1 mol dm^-3^ H_2_O_2_ as the oxidant (Figure 1f). The morphologies of the alumina mask, deposited metal layer, and etched silicon were determined by field-emission scanning electron microscopy (FE-SEM, JSM-6701 F, JEOL Ltd., Akishima-shi, Tokyo, Japan) and atomic force microscopy (AFM, Digital Instrument NanoScope IIIa, Tonawanda, NY, USA) using silicon conical tips with a typical radius of curvature of 10 nm.

## Results and discussion

### Preparation of porous alumina mask on silicon substrate

We previously reported that the transfer of a porous pattern of anodic alumina into a silicon substrate can be achieved by removing silicon oxide, which is produced by the localized anodization of the silicon substrate underneath the barrier layer of anodic alumina [20,21]. The periodicity of the hole arrays obtained on the silicon substrate, which was basically determined by the pore interval of the upper anodic porous alumina, was approximately 100 nm, corresponding to a formation voltage of 40 V. However, the hole arrays obtained were shallow concave arrays with a depth of approximately 10 nm. Here, we attempted to fabricate sub-100-nm silicon nanohole arrays with a high aspect ratio using metal-assisted chemical etching.

**Figure 2:**
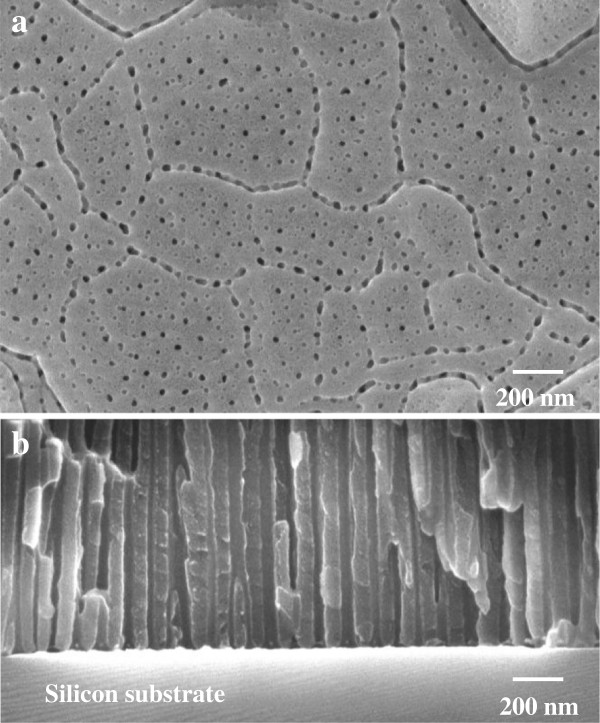
**SEM images of porous alumina mask. (a)** Surface and **(b)** cross-sectional SEM images of porous alumina mask formed on the Si substrate after anodization.

For the subsequent pattern transfer, it was essential to stop anodization at an appropriate stage when current is at its minimum in the current-time curve. The anodization behavior was described in detail in our previous reports [20,21]. When anodization was stopped at the minimum current, the morphology of the anodic porous alumina remaining on the silicon substrate was observed using SEM. On the surface, pore initiation proceeded preferentially at the grain boundary of the aluminum deposited by sputtering, as shown in Figure 2a. The top diameter of pores in the anodic alumina film was approximately 20 nm, smaller than that of the bottom part following the well-established pore initiation mechanism [23]. Although the pore arrangement was random on the film surface, the regularity of pore arrangement improved gradually in the direction of pore depth by self-ordering. After the chemical dissolution of the barrier layer in phosphoric acid, the cross section of the alumina mask was observed. As shown in Figure 2b, no barrier layer at the bottom part of each pore in the porous alumina film was observed. In other words, a through-hole alumina mask could be obtained directly on a silicon substrate by the selective removal of the barrier layer because the thickness of the barrier layer decreases by approximately half during the unique deformation of the bottom part of anodic porous alumina [24,25].

### Formation of noble metal dot arrays on silicon

**Figure 3:**
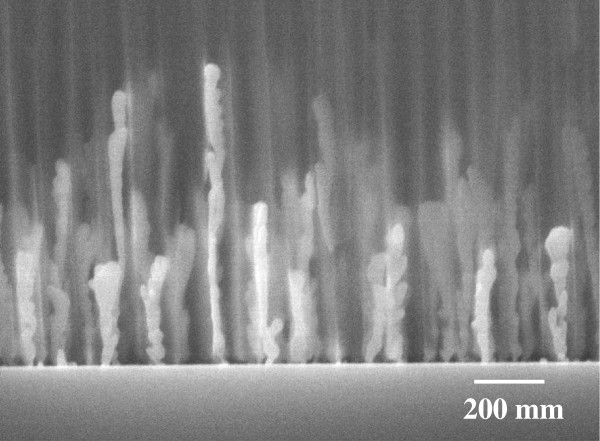
**Ag nanowire arrays formed on Si substrate.** SEM image of Ag nanowire arrays formed on Si substrate through anodic porous alumina mask. Metal deposition was conducted in a solution of 2 × 10^-2^ mol dm^-3^ AgNO_3_ and 5 mol dm^-3^ HF for 5 s.

To examine the morphology of the deposited Ag dots, electroless metal deposition was conducted under many conditions. Figure 3 shows a typical cross-sectional image of silicon with the anodic alumina mask after the immersion in 5 mol dm^-3^ HF solution containing a relatively high AgNO_3_ concentration of 2 × 10^-2^ mol dm^-3^ for 5 s. From this SEM image, it was confirmed that the Ag nanowires were grown inside the nanopores of anodic alumina mask in a direction perpendicular to the surface of silicon substrate. The periodicity of Ag nanowires, which was determined by the pore interval of the anodic alumina mask formed at 40 V, was approximately 100 nm. Note that each Ag nanowire has almost the same diameter, determined by the pore size of the alumina mask, while the length of Ag nanowires was mainly determined by the immersion time.

**Figure 4:**
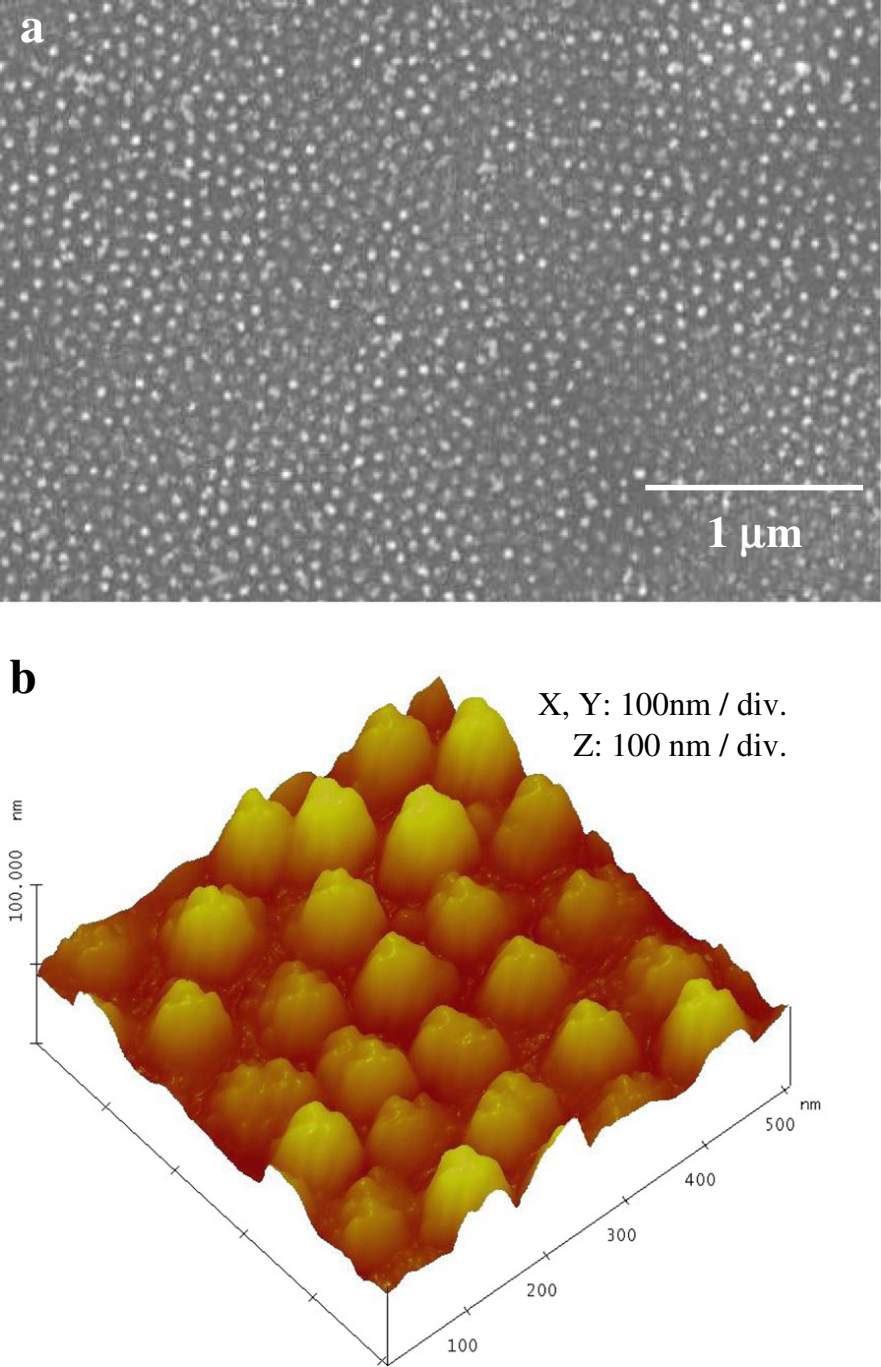
**Ag nanodot arrays formed on Si substrate. (a)** SEM image of Ag nanodot arrays formed on Si substrate through anodic porous alumina mask. **(b)** AFM tapping mode image. Metal deposition was conducted in a solution of 2 × 10^-3^ mol dm^-3^ AgNO_3_ and 5 mol dm^-3^ HF for 15 s.

By decreasing the concentration of AgNO_3_, the size of the deposited Ag dots could be optimized. After the immersion in 5 mol dm^-3^ HF solution containing 2 × 10^-3^ mol dm^-3^ AgNO_3_ for 15 s, the surface of silicon was observed using SEM. In this case, the anodic alumina film used as a mask was dissolved during the electroless deposition of Ag. Because the prolongation of deposition time caused the interlocking of the deposited Ag owing to the excessive deposition of Ag nanoparticles, the period of electroless metal deposition was standardized to 15 s. As shown in Figure 4a, well-ordered Ag nanodot arrays on the silicon substrate corresponding to the configuration of a self-organized pore arrays in the anodic alumina mask were observed. To evaluate the size of the deposited Ag dots, AFM observation was also carried out. As indicated in Figure 4b, the diameter and height of Ag dots were approximately 40 nm and approximately 20 nm, respectively. Although the regularity of the configuration of Ag nanodot arrays was not always sufficient, the regularity of these patterns is thought to be affected by the morphology and the thickness of the aluminum film deposited by sputtering as shown in Figure 2a. In general, pore arrangement of porous alumina is known as an imperfect structure. Especially, its structure shows only short-range ordering at the initial stage of anodization. Many studies demonstrate the fact that it is impossible to obtain almost perfect hexagonal pore arrangement in anodic alumina film when thin aluminum film sputtered on a solid substrate is applied as a specimen [17,20-22,24-26]. To improve the regularity of pore arrangement of porous alumina, two-step anodization [27] or nanoindentation process [28] are found to be a useful technique.

The formation of the metal dot pattern on the silicon substrate can be explained by the mechanism of displacement plating, as demonstrated in the case of copper in our previous work [26]. In this work, the electroless deposition of Ag on a silicon substrate could be achieved in a AgNO_3_/HF solution by the predominant dissolution of SiO_2_, which is produced by the localized anodization of the silicon substrate underneath the barrier layer of the upper alumina mask, and the subsequent dissolution of silicon to supply electrons for Ag deposition. On the basis of the present method, it must be noted that noble metal nanodot arrays can be formed directly and spontaneously on a silicon substrate over a large area without any dry process such as sputtering. Moreover, in principle, there is no limit to the deposition area that can be patterned because it is not necessary to use special vacuum equipment. Although the controllability of Ag deposition needs to be improved further, the proposed pattern transfer is suitable for the large-scale production of ordered noble metal dot pattern on a silicon substrate.

### Metal-assisted chemical etching of silicon using patterned metal dot arrays

**Figure 5:**
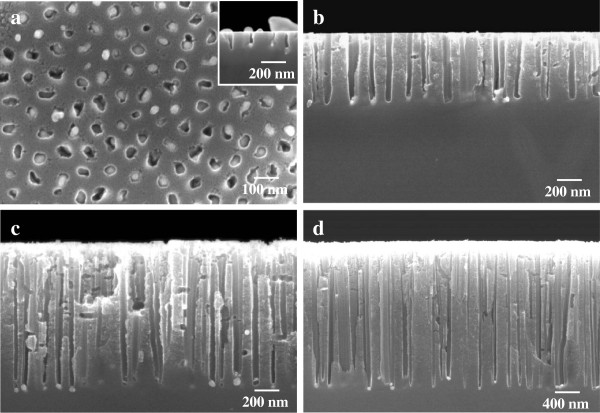
**SEM images of Si nanohole arrays fabricated by Ag-assisted chemical etching.** SEM images of Si nanohole arrays fabricated by Ag-assisted chemical etching in 5 mol dm^-3^ HF - 1 mol dm^-3^ H_2_O_2_ solution for **(a)** 20 s, **(b)** 30 s, and **(c)** 1 min. **(d)** Silicon nanohole arrays formed in 10 mol dm^-3^ HF - 1 mol dm^-3^ H_2_O_2_ solution for 1 min. (a) top and (b-d) cross-sectional SEM images.

After the formation of Ag dot arrays on the silicon substrate, the specimens were immersed in a solution of HF and H_2_O_2_ to form silicon nanohole arrays by metal-assisted chemical etching. Figure 5 shows SEM images of the etched silicon surface using the patterned Ag catalyst. The silicon nanoholes obtained were arranged hexagonally over the entire area of the specimen. When Ag nanoparticles deposited randomly without the use of mask were applied as a catalyst, the regularity of arrangement of silicon nanoholes was extremely low [29,30]. In this work, the periodicity of the silicon nanohole arrays was approximately 100 nm, corresponding to that of the Ag dot arrays used as the catalyst and that of the pores in porous alumina used as the initial mask. Ag particles, which were detected as circular bright spots, were observed inside holes in the silicon substrate, as shown in Figure 5a. The diameter of the holes observed in Figure 5a coincided with the sizes of the deposited Ag particles. These results indicate that chemical etching occurred one-to-one only at the Ag/silicon interface and proceeded anisotropically perpendicular to the substrate, i.e., in the <100> direction as shown in the inset of Figure 5a. The area of contact between the alumina mask and the underlying silicon substrate remains as a rim of the silicon nanohole at the surface of silicon.

After chemical etching for 30 s, the cross-sectional image reveals that the depth of the silicon nanoholes reached 600 nm, which is equivalent to an aspect ratio of 15 (depth divided by the hole diameter of 40 nm, as shown in Figure 5b). To form deeper hole arrays in the silicon, etching time was prolonged from 30 s to 1 min. The depth of the silicon nanohole arrays increased with increasing etching time. In the case of chemical etching for 1 min, the depth and aspect ratio of the silicon holes were approximately 1.2 μm and approximately 30, respectively (Figure 5c). The depth increased by almost twice the depth of the hole arrays is shown in Figure 5b.

**Figure 6:**
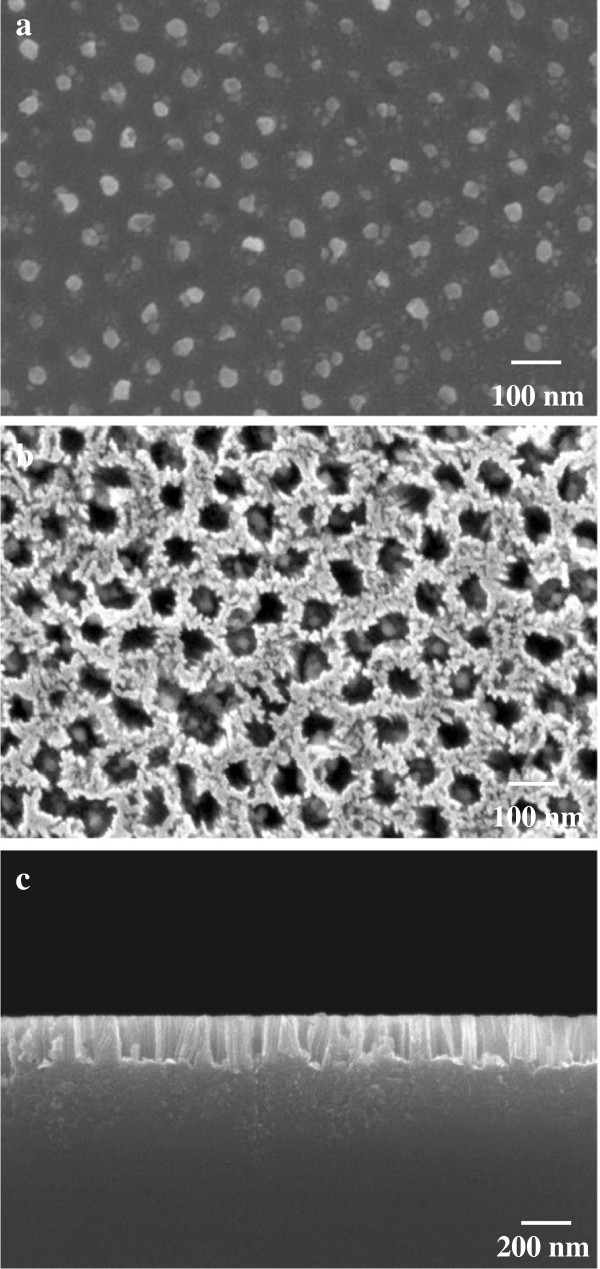
**SEM images of Si nanohole arrays fabricated by Au-assisted chemical etching. (a)** SEM image of Au nanodot arrays formed on Si substrate through anodic porous alumina mask. **(b)** Top and **(c)** cross-sectional SEM images of Si nanohole arrays fabricated by Au-assisted chemical etching in 5 mol dm^-3^ HF - 1 mol dm^-3^ H_2_O_2_ solution for 1 min.

To examine the effect of catalyst species on the morphology of etched silicon structures, chemical etching was also carried out using patterned Au nanodot arrays formed by a similar displacement plating. When the composition of the plating solution was changed from AgNO_3_/HF to Na[AuCl_4_] · 2H_2_O/HF, highly ordered Au nanodot arrays were also obtained on the silicon substrate, as shown in Figure 6a. Each dot appears to consist of two or three particles with average sizes of 20 to 40 nm. The morphology of the dots was quite similar to that of the copper dots deposited by electroless deposition in our previous work [26].

Figure 6b shows a SEM image of the etched silicon surface using the patterned Au catalyst. The surface morphology of the etched silicon was different from that of the hole arrays formed using the Ag catalyst, as shown in Figure 5. The notable features of the nanoholes formed using the Au catalyst are that the opening of holes was wider and rough around the edges at the upper part. In addition, the etching rate using the Au catalyst was significantly lower than that in the case of using the Ag catalyst even under the same etching conditions, as shown in Figure 5c. When the etching time was equal to 1 min, the depth and aspect ratio of the silicon holes were approximately 200 nm and approximately 5, respectively (Figure 6c). That is, the etching rate was six times lower for the Au catalyst than for the Ag catalyst. The reason for the difference in etching rate might be the difference in the catalytic activity of the noble metal and in the morphology of the catalyst [9,13]. Although the depth of the holes was basically determined by etching time, prolonged chemical etching in 5 mol dm^-3^ HF - 1 mol dm^-3^ H_2_O_2_ using the Au catalyst caused the formation of a tapered hole structure due to the chemical dissolution of the horizontal plane at the outermost surface by the diffusion of positive holes (h^+^). This result indicates that the diffusion of h^+^ from the metal/silicon interface was suppressed effectively when Ag was used as the catalyst, compared with Au.

Figure 5d shows the silicon straight nanohole arrays with a high aspect ratio formed using the Ag catalyst. When metal-assisted chemical etching was conducted in HF at a high concentration of 10 mol dm^-3^, the etching rate was 1.67 times higher than that in the case using a relatively low HF concentration of 5 mol dm^-3^. In the case of chemical etching for 1 min, the depth and aspect ratio of the silicon holes were approximately 2 μm and approximately 50, respectively. The aspect ratio of the silicon hole formed by metal-assisted chemical etching in this work was about ten times higher than that of the previous work using electrochemical etching through alumina mask [19]. One of the notable features of the silicon nanohole structure obtained is that the diameter of each hole hardly increased during chemical etching. In other words, the dissolution of silicon proceeded locally only at the metal/silicon interface owing to suppression of the diffusion of h^+^ in highly concentrated HF, resulting in the formation of straight nanoholes with a high aspect ratio. The effect of etchant concentration on etching rate was in good agreement with previous results [12,30].

### Reduction in hole periodicity

**Figure 7:**
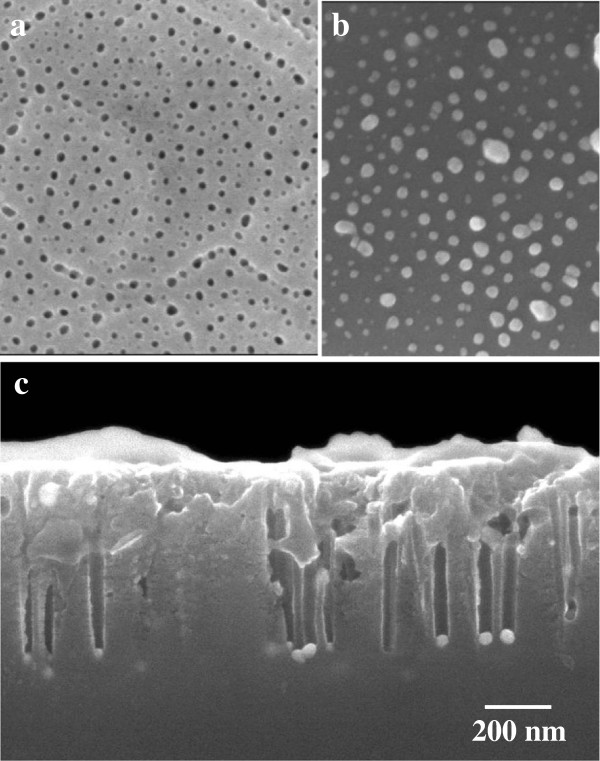
**Reduction in hole periodicity.** SEM images of **(a)** surface of porous alumina mask and **(b)** Ag nanodot arrays with 60-nm periodicity formed on Si substrate. **(c)** Cross-sectional SEM image of Si hole arrays fabricated by metal-assisted chemical etching in 5 mol dm^-3^ HF - 1 mol dm^-3^ H_2_O_2_ solution for 1 min.

The periodicity of hole arrays in a silicon substrate is basically determined by the pore interval of the upper anodic porous alumina. Here, an Al film sputtered on the silicon substrate was anodized in sulfuric acid as described previously [22]. Figure 7a shows the pore arrangement of the alumina mask at the film surface. The pore interval was shorter than that of the alumina shown in Figure 2a. To prepare Ag nanodot patterns on the silicon substrate, the anodized specimen was immersed in a solution of AgNO_3_ and HF solutions, as described above. After metal deposition for 15 s, the surface of the silicon substrate was observed using SEM. Figure 7b shows Ag nanodot arrays on the silicon substrate corresponding to the configuration of self-organized pore arrays in the anodic alumina mask. The periodicity and diameter of the Ag dots were approximately 60 nm and approximately 30 nm, respectively.

Figure 7c shows silicon nanohole arrays with a reduced hole periodicity of 60 nm. The periodicity of the nanoholes obtained decreased to 60% of that shown in Figure 5 because of the reduction in formation voltage for the alumina mask from 40 to 25 V. After chemical etching for 1 min, the diameter and depth of the nanoholes were approximately 30 nm and approximately 540 nm, respectively. The estimated aspect ratio was approximately 18, which was lower than that shown in Figure 5c. Although catalyst types (Ag) and etching times (1 min) were the same in both cases, hole depth, in other words, etching rate, decreased. At the periodicity of 60 nm shown in Figure 7, the deposited Ag particles were smaller than those at the periodicity of 100 nm, as shown in Figure 5, because of the reduction in the opening area of the alumina mask used for metal deposition. Consequently, suppressing the catalytic reaction, which has direct effects on anodic oxidation and silicon dissolution, was considered.

A similar phenomenon related to the relationship between etching rate and the amount of catalyst was also reported by other groups [31,32]. Lee et al. demonstrated that the fast etching rate for the aggregated spherical Au particles (particle sizes of approximately 1 μm) was attributable to the larger surface area of Au catalyst [31]. When the amount of reduction of H_2_O_2_ per unit area of the cross section of the holes increases, the number of h^+^ injected into silicon should increase. As a result, it is concluded that the etching rate increases with an increase of the area of the catalyst. In other words, the total volume of the silicon dissolved during metal-assisted chemical etching strongly correlates with the area of the catalyst. In this work, it is notable that catalyst size effect was confirmed even when nanometer-sized metal particles were applied as catalysts. In addition, investigation of the effect of metal catalysts on the morphology of etched silicon using ordered arrays of size-controlled catalysts is thought to be significant from the perspective of development of precise nanofabrication methods of semiconductors.

## Conclusions

In summary, a resist-free nonlithographic method for the fabrication of ordered silicon nanohole arrays by a combination of localized metal deposition and the subsequent metal-assisted chemical etching was demonstrated. The porous alumina formed directly on the Si substrate served as a mask for localized metal deposition and controlled the position and size of noble metals, which were deposited only in the exposed area at the alumina mask/silicon interface. After metal deposition, the pattern transfer of the self-ordered pore configuration of porous alumina into silicon was examined by metal-assisted chemical etching. In brief, the present process consists of two independent processes: (1) noble metal nanodot arrays are obtained by displacement plating using an alumina mask in HF solution containing the desired metal ion and (2) straight silicon nanohole arrays are formed by the site-selective etching of silicon using the deposited noble metal as the catalyst in a solution of HF and H_2_O_2_. The dimensions of the resultant nanohole pattern can be controlled by changing the anodization conditions of aluminum for forming an alumina mask, which include electrolyte type and anodization voltage, and the chemical etching conditions such as catalyst type, catalyst amount, etchant concentration, and etching time. The periodicity of silicon hole arrays, which was basically determined by the pore interval of the upper anodic porous alumina, could be adjusted to 60 and 100 nm, corresponding to formation voltages of 25 and 40 V, respectively.

## Abbreviations

AFM: Atomic force microscopy; SEM: Scanning electron microscope.

## Competing interests

The authors declare that they have no competing interests.

## Authors’ contributions

HA and SO conceived the idea and designed the experiments. KF carried out all the experiments and data analysis under the instruction of SO. All the authors contributed to the preparation and revision of the manuscript and read and approved its final version.

## Authors’ information

HA is an associate professor, KF is a graduate student, and SO is a professor at the Department of Applied Chemistry, Kogakuin University.
